# Effects of Cybersickness Caused by Head-Mounted Display–Based Virtual Reality on Physiological Responses: Cross-sectional Study

**DOI:** 10.2196/37938

**Published:** 2022-10-17

**Authors:** Yoon Sang Kim, JuHye Won, Seong-Wook Jang, Junho Ko

**Affiliations:** 1 BioComputing Lab, Institute for Bio-engineering Application Technology Department of Computer Science and Engineering Korea University of Technology and Education Cheonan-si Republic of Korea; 2 BioComputing Lab Department of Computer Science and Engineering Korea University of Technology and Education Cheonan-si Republic of Korea; 3 Assistive Technology Research Team for Independent Living National Rehabilitation Institute Seoul Republic of Korea; 4 AirPlug Ltd Seoul Republic of Korea

**Keywords:** cybersickness, physiological responses, virtual reality, VR, head-mounted displays, heart rate, cortisol

## Abstract

**Background:**

Although more people are experiencing cybersickness due to the popularization of virtual reality (VR), no official standard for the cause and reduction of cybersickness exists to date. One of the main reasons is that an objective method to assess cybersickness has not been established. To resolve this, research on evaluating cybersickness with physiological responses that can be measured in real time is required. Since research on deriving physiological responses that can assess cybersickness is at an early stage, further studies examining various physiological responses are needed.

**Objective:**

This study analyzed the effects of cybersickness caused by head-mounted display–based VR on physiological responses.

**Methods:**

We developed content that provided users with a first-person view of an aircraft that moved (with translation and combined rotation) over a city via a predetermined trajectory. In the experiment, cybersickness and the physiological responses of participants were measured. Cybersickness was assessed by the Simulator Sickness Questionnaire (SSQ). The measured physiological responses were heart rate, blood pressure, body temperature, and cortisol level.

**Results:**

Our measurement confirmed that all SSQ scores increased significantly (all *P*s<.05) when participants experienced cybersickness. Heart rate and cortisol level increased significantly (*P*=.01 and *P*=.001, respectively). Body temperature also increased, but there was no statistically significant difference (*P*=.02). Systolic blood pressure and diastolic blood pressure decreased significantly (*P*=.001).

**Conclusions:**

Based on the results of our analysis, the following conclusions were drawn: (1) cybersickness causes significant disorientation, and research on this topic should focus on factors that affect disorientation; and (2) the physiological responses that are suitable for measuring cybersickness are heart rate and cortisol level.

## Introduction

The recent development of technologies such as head-mounted displays (HMDs) and motion-tracking devices has enabled active research on virtual reality (VR). VR is used in various fields, such as in games, education, medicine, and health care [1]. Although VR can improve the user’s concentration by providing an immersive experience, some users may experience cybersickness, which is a type of motion sickness [2].

The most well-known theory for explaining motion sickness is the sensory conflict theory. According to the sensory conflict theory, motion sickness is caused by a discordance between the vestibular sense and the visual perception of body movement [3-6]. In addition, according to the sensory conflict theory, motion sickness is classified into motion-induced motion sickness (MIMS) and visually induced motion sickness (VIMS). MIMS is further classified according to the external environment as car, ship, and air (flight) sickness [7-10], and VIMS is further classified into simulator motion sickness and cyber motion sickness according to the display device [11,12]. Simulator motion sickness is caused in virtual training such as flying and driving [13-15], and cyber motion sickness is caused in virtual environments that are completely different from real ones [16].

Although more people are experiencing cybersickness due to the popularization of VR, no official standard for the cause and reduction of cybersickness exists to date. One of the main reasons is that an objective method to assess cybersickness has not been established. Vomiting has been used as a diagnostic criterion for motion sickness because it is difficult to assess other symptoms quantitatively [17]. However, because there are instances of motion sickness that do not accompany vomiting, the Motion Sickness Assessment Questionnaire (MSAQ) [18] and the Simulator Sickness Questionnaire (SSQ) [19] have been proposed to assess motion sickness symptoms. The MSAQ has been used extensively in traditional motion sickness studies; however, it is not suitable for VIMS assessment. The SSQ, which is optimized from the MSAQ and focuses on VIMS, is mostly used in studies assessing simulator sickness and cybersickness. Even though the SSQ is low-cost and easy to use, its objectiveness is questionable and its real-time implementation is difficult [20,21].

To solve this problem, research on evaluating cybersickness with physiological responses that can be measured in real time is required [22,23]. To assess cybersickness in terms of physiological responses, it is necessary to find out the factors that are correlated with cybersickness. Conventional studies have reported that physiological responses, such as specific frequency power bands of the electroencephalogram [24-26], gastrointestinal activity [27], heart rate [28-30], and skin conductance [31], can be used to assess cybersickness. In particular, heart rate has been reported to be related to the stress task when playing a video [29,30]. Although conventional research into cybersickness is extensive, this issue remains in the HMD-based VR context [32]. Many validation experiments are necessary to generalize these physiological responses. Since research on deriving physiological responses that can assess cybersickness is at an early stage, further research on various physiological responses is still required. Therefore, this paper deals with the effects of cybersickness caused by HMD-based VR on physiological responses.

## Methods

### Design and Setting

**Figure 1:**
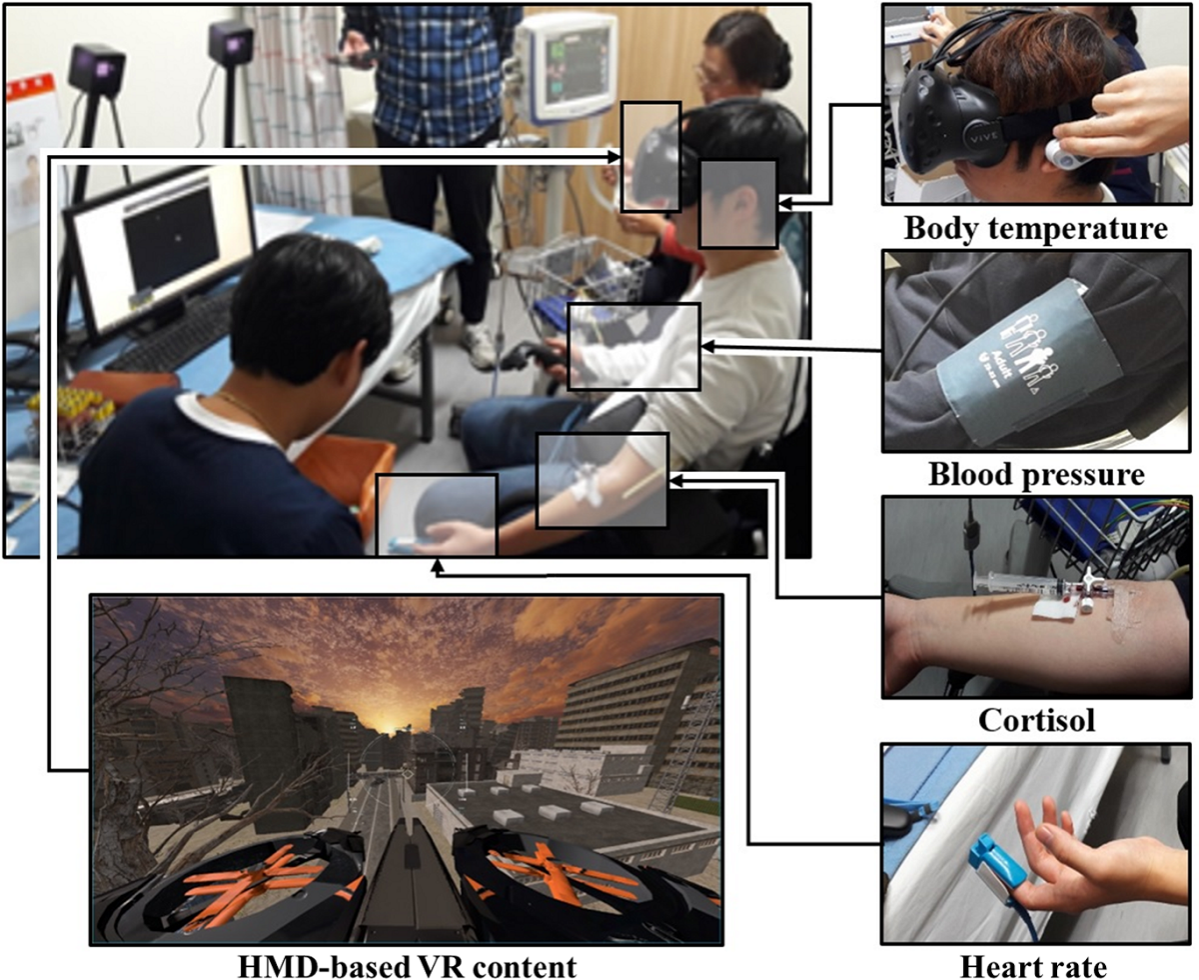
The experimental environment. HMD: head-mounted display; VR: virtual reality.

We performed an experiment where participants watched HMD-based VR content in the environment shown in Figure 1.

Conventional studies on the effect of the content itself have shown that rotational movement causes higher motion sickness than a linear one [33] and that a combined rotation of more than 1 axis causes greater motion sickness than a rotation of a single axis [34,35]. Based on those conventional studies, our HMD-based VR content was developed to intentionally cause cybersickness using Unity 3D (Unity Technologies) [36]. In addition, the longer the exposure time, the higher the level of cybersickness, so the playing time of the developed content was configured to be the least amount of time needed to measure the physiological response.

The developed content provided the user with a first-person view of an aircraft that moved (with translation and combined rotation) over a city via a predetermined trajectory.

### Questionnaire, Variables, and Equipment

The SSQ [19] was used to assess cybersickness. This questionnaire consists of 16 questions with 3 subscales corresponding to symptom clusters (nausea, oculomotor symptoms, and disorientation). Each question is measured on a 4-point scale from 0 to 3 points. A total score represents the complete symptom level of motion sickness. A higher score indicates more severe motion sickness.

In an experiment conducted on a Korean population [37], it was confirmed that the SSQ significantly increased in HMD-based environments compared to screen-based environments, which suggests it could have validity and reliability in measuring cybersickness.

The occurrence of motion sickness is highly related to the autonomic nervous system [38]. Therefore, in this experiment, physiological responses related to the autonomic nervous system were measured to examine whether or not the responses related to motion sickness can be applied to HMD-based cybersickness. We measured the following physiological responses that relate to the autonomic nervous system: heart rate, blood pressure, body temperature, and cortisol level. Blood pressure was measured as 2 values: systolic and diastolic. Systolic blood pressure indicates the highest pressure in the artery when the heart is contracted, and diastolic blood pressure indicates the lowest pressure in the artery right before the heart contracts again. Body temperature was measured with a digital thermometer. Cortisol, which is produced under stress, was measured from 4 cc of blood collected over 2 minutes with the support of a clinician at Cheonan Medical Center [39].

The HMD used in the experiment was the HTC Vive (HTC Corporation) [40]. To minimize the effect of the vestibular sense, we controlled body motion in addition to head translation and rotation.

### Participants

A total of 16 undergraduate and graduate students (male: n=8, 50%; female: n=8, 50%) participated in the experiment. The participants had no history of problems associated with the nervous system, autonomic nervous system, and visual system. In addition, more than 8 hours of sleep was recommended to prevent increased cybersickness sensitivity among participants [41]. Prior to participation, we explained the experiment, apart from the objective, and then obtained written consent to participate.

### Procedures

The experimental procedure consisted of 3 steps (pre-experiment, experiment, and postexperiment) as shown in Textbox 1. In the pre-experiment step, SSQ and the physiological responses of the participants were measured. 

In the experiment step, participants viewed the HMD-based VR content that we developed during the design phase. The participants sat and watched the VR content without moving. By making the participants concentrate only on watching the VR content, we ensured their stress level was affected solely by cybersickness.

In the postexperiment step, participants’ physiological responses and SSQ outcomes were measured. Each step was performed for 2 minutes, and the total experiment time was 10 minutes.

**The experimental procedure.**

**Pre-experiment step**

- Simulator Sickness Questionnaire (SSQ) measurement (2 minutes)
- Physiological response measurement (2 minutes)

**Experiment**

- Head-mounted display–based virtual reality content viewing (2 minutes)

**Postexperiment step**

- Physiological response measurement (2 minutes)
- SSQ measurement (2 minutes)

### Data Analysis

We analyzed the data measured in the experiment using SPSS Statistics (version 21; IBM Corp) [42]. The data measured in the experiment were SSQ items, heart rate, body temperature, blood pressure, and cortisol. A statistical analysis (paired *t* test) was applied to find significant differences between the measured data in the pre- and postexperiment steps. SSQ scores from before and after the experiment were compared to detect the presence of cybersickness. If found, we analyzed how each physiological response was related to cybersickness in the HMD-based environment.

### Ethics Approval

The study was approved by the institutional review board of the Korea University of Technology and Education (IRB-17122602).

## Results

### SSQ Analysis

**Figure 2:**
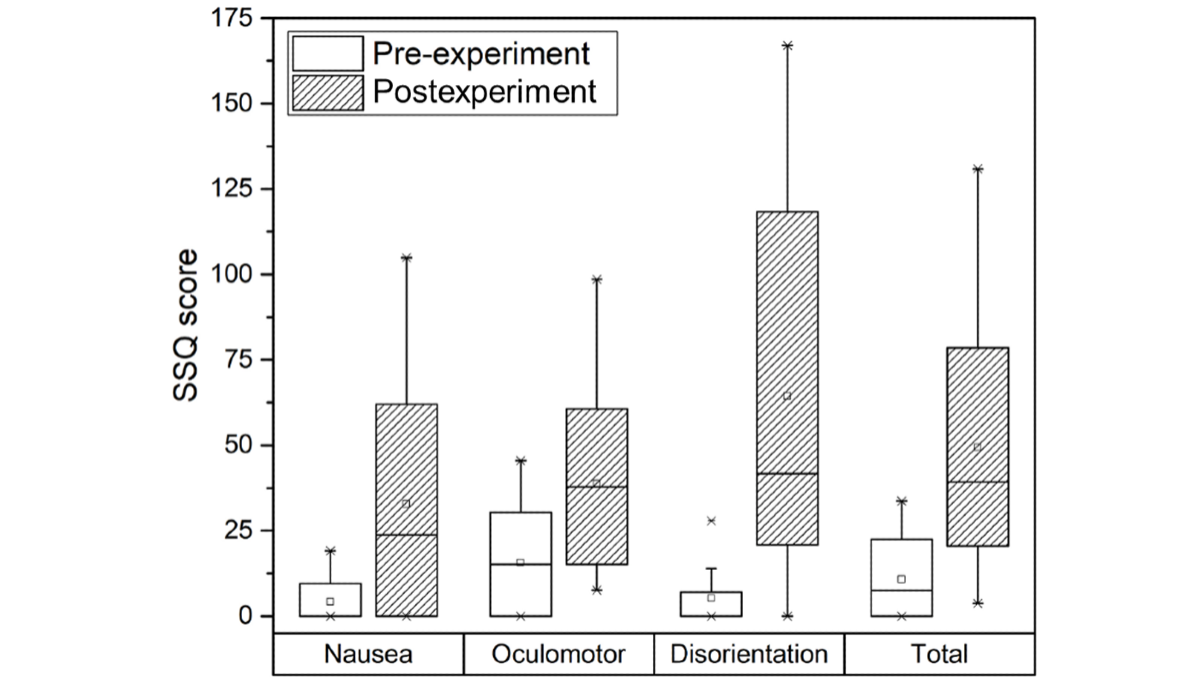
The SSQ scores measured in the pre- and postexperiment steps. SSQ: Simulator Sickness Questionnaire.

The SSQ scores measured before and after the experiment are shown in Figure 2. In the pre-experiment step, the mean SSQ scores for nausea, oculomotor, disorientation, and total score were 4.17, 15.63, 5.22, and 10.75, respectively. In the postexperiment step, they were 32.79, 38.77, 64.38, and 49.32, respectively. From the measurement results, it was confirmed that all SSQ scores increased significantly, as shown in Table 1 (all *P*s<.05). This indicates that cybersickness was experienced by participants viewing the HMD-based VR content.

**Table 1:** The Simulator Sickness Questionnaire scores measured before and after the experiment.

Physiological response	Pre-experiment score, mean (SD)	Postexperiment score, mean (SD)	*t* (*df*)	*P* value
Nausea	4.17 (6.94)	32.79 (34.30)	–3.48 (15)	.003^a^
Oculomotor	15.63 (16.01)	38.77 (24.97)	–3.37 (15)	.004^a^
Disorientation	5.22 (10.01)	64.38 (58.15)	–3.93 (15)	.001^a^
Total	10.75 (11.74)	49.32 (38.00)	–3.89 (15)	.001^a^

^a^Significant *P* values.

### Physiological Responses Analysis

**Figure 3:**
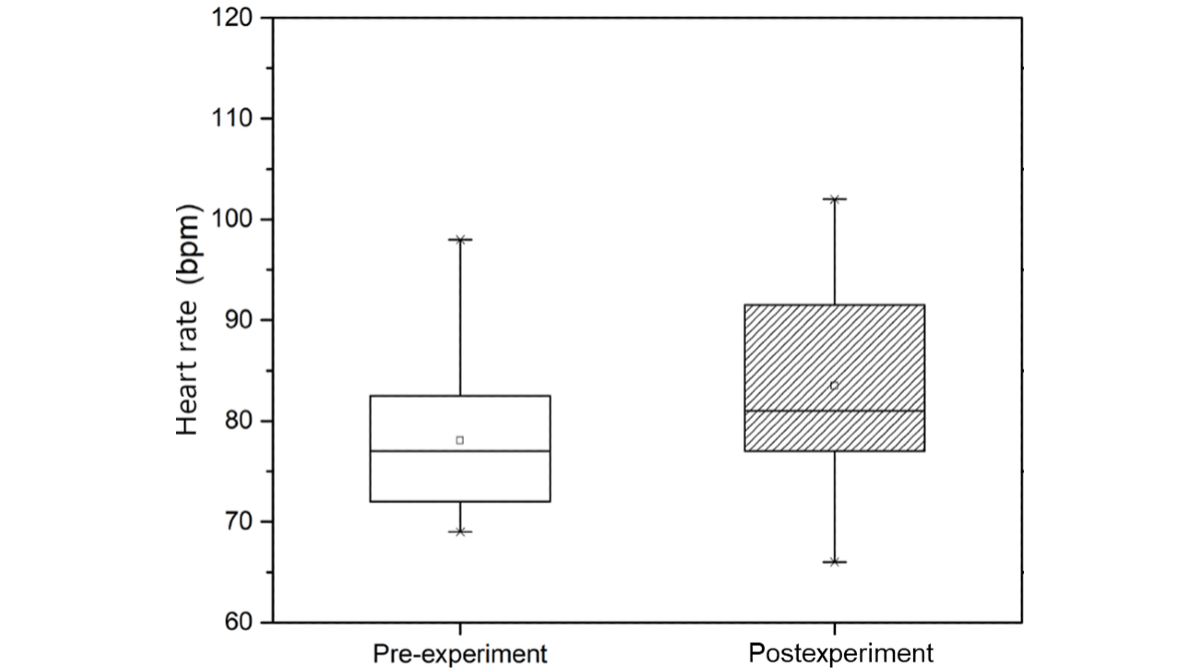
Heart rate measured in the pre- and postexperiment steps.

The physiological responses measured in the pre- and postexperiment steps are shown in Table 2. Mean heart rate before and after the experiment was 78.06 bpm and 83.50 bpm, respectively. Our measurements confirmed that heart rate increased significantly (*P*=.01; Figure 3). This means that heart rate increased when cybersickness was experienced by participants viewing the HMD-based VR content.

**Table 2:** Physiological responses measured before and after the experiment.

Physiological response		Pre-experiment score, mean (SD)	Postexperiment score, mean (SD)	*t* (*df*)	*P* value
Heart rate (bpm)		78.06 (7.71)	83.50 (10.41)	–2.92 (15)	.01^a^
Cortisol (ug/dl)		7.75 (2.62)	10.59 (4.12)	–4.72 (15)	.001^a^
Body temperature (°C)		37.10 (0.28)	37.14 (0.21)	–1.33 (15)	.20
**Blood pressure (mmHg)**					
	Systolic	130.81 (18.84)	117.31 (15.78)	5.43 (15)	.001^a^
	Diastolic	76.69 (9.30)	67.50 (11.90)	4.43 (15)	.001^a^

^a^Significant *P* values.

**Figure 4:**
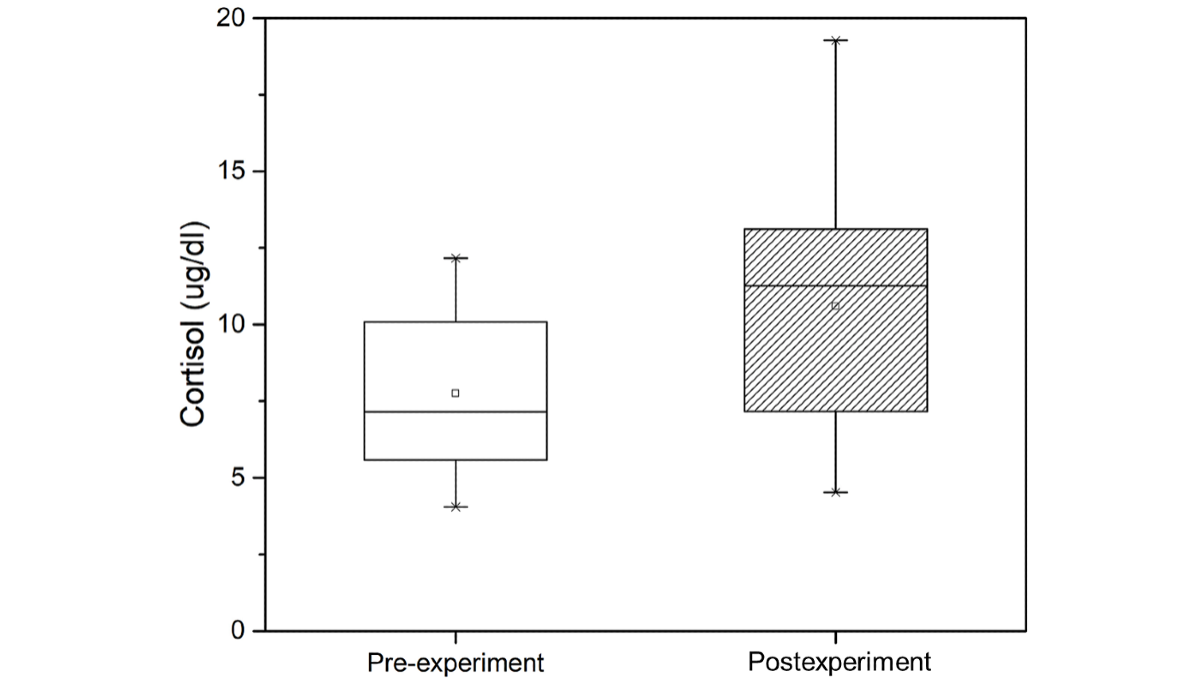
Cortisol level measured in the pre- and postexperiment steps.

The mean cortisol level before and after the experiment was 7.75 ug/dl and 10.59 ug/dl, respectively. Our measurements confirmed that the cortisol level increased significantly (*P*=.001; Figure 4). This implies that the cortisol level increased when participants felt cybersickness.

**Figure 5:**
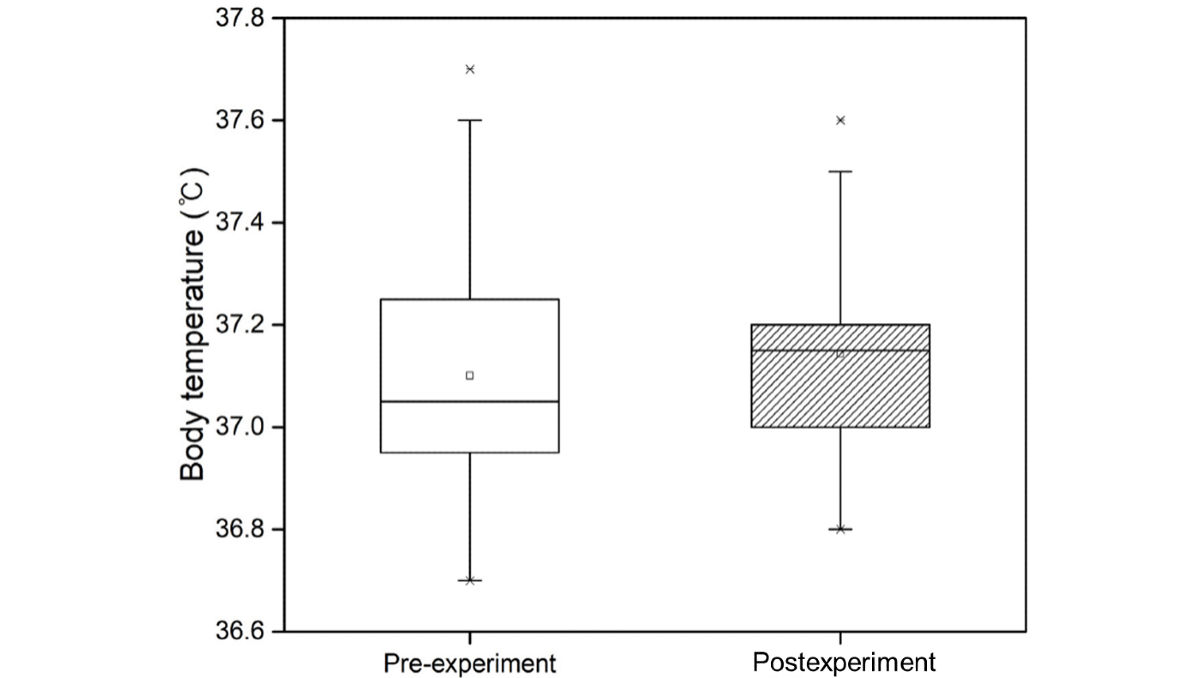
Body temperature measured in the pre- and postexperiment steps.

Mean body temperature before and after the experiment was 37.10 °C and 37.14 °C, respectively. As per the measurement results, body temperature increased (Figure 5), but there was no statistically significant difference (*P*=.20).

**Figure 6:**
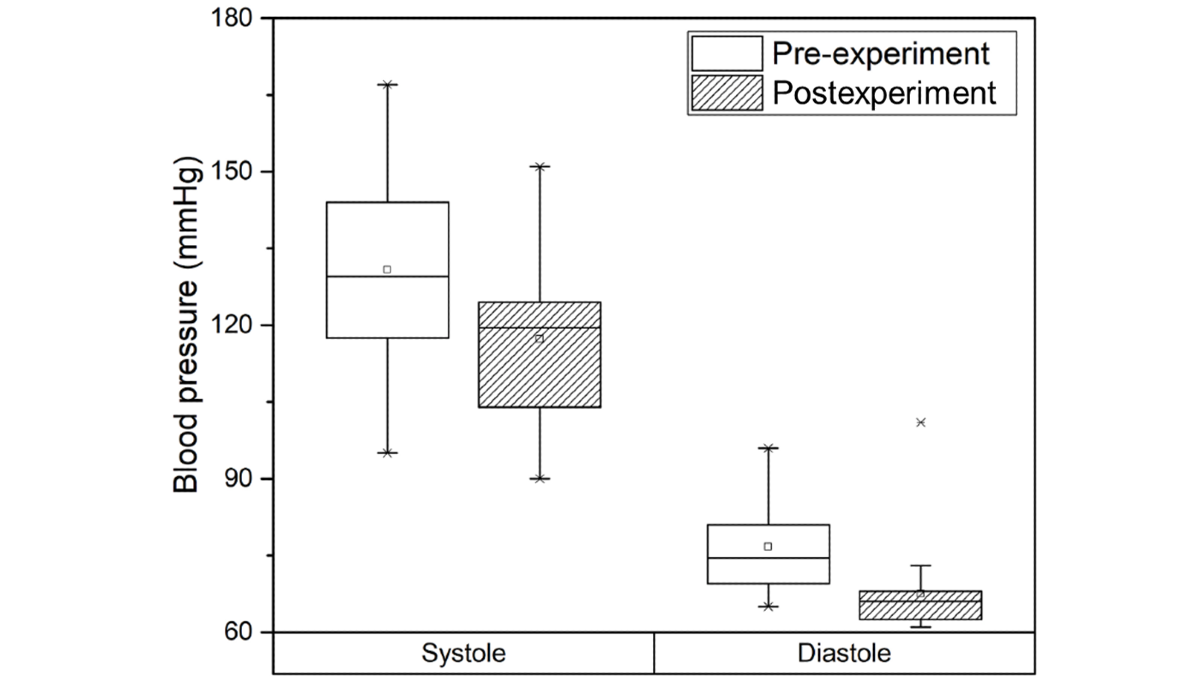
Blood pressure measured in the pre- and postexperiment steps.

Mean systolic blood pressure before and after the experiment was 130.81 mmHg and 117.31 mmHg, respectively, whereas mean diastolic blood pressure was 76.69 mmHg and 67.50 mmHg, respectively. As per the measurement results, systolic blood pressure and diastolic blood pressure decreased significantly (*P*=.001; Figure 6). This means that blood pressure decreased when cybersickness was experienced by participants.

## Discussion

### Principal Findings

This paper examined the effects of cybersickness on physiological responses when people watch HMD-based VR content. To this end, we performed statistical analyses of SSQ scores and physiological responses (using questionnaire responses and measurements, respectively) before and after the experiment.

SSQ scores analysis in the pre- and postexperiment steps was performed to assess whether HMD-based VR content caused cybersickness. According to previous studies assessing various motion sicknesses [21], MIMS (car, ship, and airplane), simulator sickness, and cybersickness showed the highest increase in nausea, oculomotor, and disorientation, respectively. In this study, we found the highest increase in disorientation in cases of cybersickness. This result matches that of other studies (see Table 1 and Figure 2). This indicates that when there is no vestibular stimulation caused by the movement of the body and cybersickness is caused by visual stimulation through the HMD, significant disorientation occurs. This means that there is a secondary risk of walking accidents due to disorientation as well as a primary risk of cybersickness when watching HMD-based VR content. Therefore, we believe that studies on reducing cybersickness should focus on disorientation.

Physiological responses analysis in the pre- and postexperiment steps was performed to assess physiological response due to cybersickness. Significant effects on heart rate, blood pressure, and cortisol level were found in participants experiencing cybersickness (Table 2). Heart rate and cortisol level are closely related to stress. Heart rate is explained separately by the heart-body linkage hypothesis and the heart-body dissociation hypothesis [43]. The heart-body connection hypothesis states that when a person exercises, their metabolism increases, which in turn causes their heart rate to increase. The heart-body dissociation hypothesis explains more reasonably the presence of cybersickness in a motionless state than the heart-body connection hypothesis. The heart-body dissociation hypothesis states that when a person is under stress, their metabolism increases and their heart rate increases proportionally. Conventional studies [29,30] on heart rate and stress found that participants’ heart rates increased when watching a video that was assigned as a stress task. In this study, we found increased heart rate (Figure 3) and cortisol level (Figure 4) in participants experiencing cybersickness. We believe that cybersickness is accompanied by stress, and heart rate increases due to the accompanying stress, which confirms the findings of previous studies. In general, it is widely known that blood pressure (Figure 5) increases as heart rate increases. However, in our study, blood pressure decreased when heart rate increased. This is because the central nervous system causes a blood pressure response [3-6] since motion sickness is an abnormal adaptation of the autonomic nervous system due to the discordance between the vestibular sense and visual perception in the central nervous system [44,45]. Therefore, we believe that the change in blood pressure that is caused by cybersickness is different from the general correlation between heart rate and blood pressure. In summary, we found that the physiological responses that can be objectively measured to indicate cybersickness are heart rate and cortisol level.

### Limitations

The number of participants (n=16) was too small to generalize the physiological responses statistically, even though they were appropriate for detecting physiological responses to cybersickness. Cybersickness is known to be sensitive to gender, age, and VR adaptation (number of experiences). However, this study did not consider these variables because our purpose was to find the physiological responses that can measure cybersickness objectively among various physiological responses. Subsequent studies should consider the number of experiences, gender, age, and VR adaptation of participants in addition to the physiological responses that were found in this study.

### Conclusions

We analyzed the effects of cybersickness caused by HMD-based VR content on physiological responses. Heart rate, body temperature, cortisol level, and blood pressure were measured to analyze SSQ scores and physiological responses. A total of 16 participants watched the HMD-based VR content in a seated position and without moving to ensure their concentration was only on watching the VR content, which was developed to intentionally cause cybersickness.

From the results of our analysis, the following conclusions were drawn: (1) cybersickness causes significant disorientation, and research on cybersickness should focus on factors that affect disorientation; and (2) the physiological responses that are suitable for measuring cybersickness are heart rate and cortisol. This means that heart rate and cortisol level can be used as real-time factors to objectively assess cybersickness.

## Abbreviations

HMD: head-mounted display
MIMS: motion-induced motion sickness
MSAQ: Motion Sickness Assessment Questionnaire
SSQ: Simulator Sickness Questionnaire
VIMS: visually induced motion sickness
VR: virtual reality

## Data Availability

All data generated or analyzed during this study are included in this published article.

## References

[1] Benzeroual KR, Allison RS (2013). Cyber (motion) sickness in active stereoscopic 3D gaming.

[2] Rebenitsch L, Owen C (2016). Review on cybersickness in applications and visual displays. Virtual Real.

[3] Oman CM (1990). Motion sickness: a synthesis and evaluation of the sensory conflict theory. Can J Physiol Pharmacol.

[4] Reason J (1978). Motion sickness: Some theoretical and practical considerations. Appl Ergon.

[5] Reason JT (2018). Motion sickness adaptation: a neural mismatch model. J R Soc Med.

[6] Reason JT, Brand JJ (1975). Motion Sickness.

[7] O'Hanlon JF, McCauley ME (1974). Motion sickness incidence as a function of the frequency and acceleration of vertical sinusoidal motion. Aerosp Med.

[8] Lawther A, Griffin M J (1988). Motion sickness and motion characteristics of vessels at sea. Ergonomics.

[9] Bos J, Bles W (1998). Modelling motion sickness and subjective vertical mismatch detailed for vertical motions. Brain Res Bull.

[10] Wertheim A, Bos J, Bles W (1998). Contributions of roll and pitch to sea sickness. Brain Res Bull.

[11] Kennedy RS, Drexler J, Kennedy RC (2010). Research in visually induced motion sickness. Appl Ergon.

[12] Naqvi SAA, Badruddin N, Malik AS, Hazabbah W, Abdullah B (2013). Does 3D produce more symptoms of visually induced motion sickness?.

[13] Miller JW, Goodson JE (1960). Motion sickness in a helicopter simulator. Aerosp Med.

[14] Crowley JS (1987). Simulator sickness: a problem for army aviation. Aviat Space Environ Med.

[15] Lerman Y, Sadovsky G, Goldberg E, Kedem R, Peritz E, Pines A (1993). Correlates of military tank simulator sickness. Aviat Space Environ Med.

[16] Mazloumi Gavgani A, Walker FR, Hodgson DM, Nalivaiko E (2018). A comparative study of cybersickness during exposure to virtual reality and "classic" motion sickness: are they different?. J Appl Physiol (1985).

[17] Shupak A, Gordon CR (2006). Motion sickness: advances in pathogenesis, prediction, prevention, and treatment. Aviat Space Environ Med.

[18] Gianaros PJ, Muth ER, Mordkoff JT, Levine ME, Stern RM (2001). A questionnaire for the assessment of the multiple dimensions of motion sickness. Aviat Space Environ Med.

[19] Kennedy RS, Lane NE, Berbaum KS, Lilienthal MG (1993). Simulator Sickness Questionnaire: an enhanced method for quantifying simulator sickness. Int J Aviat Psychol.

[20] McCauley ME, Sharkey TJ (1992). Cybersickness: perception of self-motion in virtual environments. Presence (Camb).

[21] Kennedy RS, Lane NE, Lilienthal MG, Berbaum KS, Hettinger LJ (1992). Profile analysis of simulator sickness symptoms: application to virtual environment systems. Presence (Camb).

[22] Davis S, Nesbitt K, Nalivaiko E (2014). A systematic review of cybersickness. Proceedings of the 2014 Conference on Interactive Entertainment.

[23] Weech S, Kenny S, Barnett-Cowan M (2019). Presence and cybersickness in virtual reality are negatively related: a review. Front Psychol.

[24] Chen Y, Duann J, Chuang S, Lin C, Ko L, Jung T, Lin C (2010). Spatial and temporal EEG dynamics of motion sickness. Neuroimage.

[25] Min B, Chung S, Min Y, Sakamoto K (2004). Psychophysiological evaluation of simulator sickness evoked by a graphic simulator. Appl Ergon.

[26] Arsalan Naqvi Syed Ali, Badruddin N, Jatoi MA, Malik AS, Hazabbah W, Abdullah B (2015). EEG based time and frequency dynamics analysis of visually induced motion sickness (VIMS). Australas Phys Eng Sci Med.

[27] Dennison MS, Wisti AZ, D’Zmura M (2016). Use of physiological signals to predict cybersickness. Displays.

[28] Holmes SR, Griffin MJ (2001). Correlation between heart rate and the severity of motion sickness caused by optokinetic stimulation. J Psychophysiol.

[29] Glass DC, Krakoff LR, Contrada R, Hilton WF, Kehoe K, Mannucci EG, Collins C, Snow B, Elting E (1980). Effect of harassment and competition upon cardiovascular and plasma catecholamine responses in type A and type B individuals. Psychophysiology.

[30] Turner JR, Carroll D, Courtney H (1983). Cardiac and metabolic responses to "space invaders": an instance of metabolically-exaggerated cardiac adjustment?. Psychophysiology.

[31] Gavgani AM, Nesbitt KV, Blackmore KL, Nalivaiko E (2017). Profiling subjective symptoms and autonomic changes associated with cybersickness. Auton Neurosci.

[32] Rangelova S, Motus D, André E, Ahram T (2019). Cybersickness among gamers: an online survey. Advances in Intelligent Systems and Computing.

[33] Lo W, So RH (2001). Cybersickness in the presence of scene rotational movements along different axes. Appl Ergon.

[34] Bonato F, Bubka A, Palmisano S, Phillip D, Moreno G (2008). Vection change exacerbates simulator sickness in virtual environments. Presence (Camb).

[35] Keshavarz B, Hecht H (2011). Axis rotation and visually induced motion sickness: the role of combined roll, pitch, and yaw motion. Aviat Space Environ Med.

[36] Unity3D. Unity Technologies.

[37] Jeeheon R, Seungbeon Y (2016). The effects of head mounted display and treadmill on cyber sickness in the immersive virtual reality learning environment. The Korea Educational Review.

[38] Muth ER (2006). Motion and space sickness: intestinal and autonomic correlates. Auton Neurosci.

[39] Cheonan Medical Center.

[40] Vive. HTC Corporation.

[41] Kaplan J, Ventura J, Bakshi A, Pierobon A, Lackner JR, DiZio P (2017). The influence of sleep deprivation and oscillating motion on sleepiness, motion sickness, and cognitive and motor performance. Auton Neurosci.

[42] SPSS software. IBM Corporation.

[43] Lee IH (1997). Psychophysiology.

[44] Balaban CD (1999). Vestibular autonomic regulation (including motion sickness and the mechanism of vomiting). Curr Opin Neurol.

[45] Balaban CD (2004). Projections from the parabrachial nucleus to the vestibular nuclei: potential substrates for autonomic and limbic influences on vestibular responses. Brain Res.

